# That's a wrap – the use of an Esmarch bandage to treat compartment syndrome of the forearm in a paediatric patient

**DOI:** 10.1016/j.tcr.2025.101202

**Published:** 2025-05-26

**Authors:** Ben Murphy, Patrick Carroll, Jacques Noel

**Affiliations:** a Department of Paediatric Orthopaedic Surgery, Our Lady's Children's Hospital, Crumlin, Dublin 12, Ireland; b Discipline of Surgery, Royal College of Surgeons in Ireland, 123 St Stephen's Green, Dublin 2, Ireland

**Keywords:** Compartment syndrome, Fasciotomy, Esmarch bandage, Distal radius fracture, Paediatric forearm fracture, Trauma

## Abstract

A 13 year old boy presented to our emergency department after a fall from his bicycle and sustained a left radius & ulna fracture. The boy had paraesthesia and reduced sensation in his digits. AIN and PIN were intact. He underwent MUA & casting in theatre. He had significant swelling, paraesthesia and severe pain with passive movement of his digits post-op. A diagnosis of compartment syndrome with suspected acute carpal tunnel syndrome was made. Flexor compartment pressure was 68 mmHg and 16 mmHg in the extensor compartment. An Esmarch bandage was temporarily applied in a retrograde fashion from distal to proximal on the elevated limb. The technique was repeated 4 times and final flexor compartment pressure was 23 mmHg. Based on these measurements, carpal tunnel release and distal radius fixation was performed, but no fasciotomy. He remained asymptomatic throughout follow-up and was subsequently discharged from the fracture clinic. We have described a successful case of treating forearm compartment syndrome in the setting of a paediatric forearm fracture conservatively, without the need for a fasciotomy. We demonstrated an objective improvement in compartment pressures with repeated applications of the Esmarch bandage technique. It is quick to implement and safe for the patient. We advocate its use in those patients where a fasciotomy is already planned as that remains the gold standard treatment. This technique should be used to potentially avoid a fasciotomy and the subsequent morbidity associated with that surgical procedure. It should be used in conjunction with sound clinical judgment and examination technique.

## Introduction

Compartment syndrome of an extremity is often associated with direct trauma and underlying fracture. It is a surgical emergency and requires vigilance to ensure its prompt diagnosis and treatment. Typical treatment for a suspected compartment syndrome involves urgent surgical decompression in the form of a fasciotomy. This is performed to prevent ischemic or neurological injury. Elevation of the affected limb is often attempted in the early stages to allow for some passive drainage of fluid from the affected compartment, and perhaps negate the need for a fasciotomy. This procedure, although effective for treating compartment syndrome, can be associated with significant morbidity. Fasciotomy incisions are generally not closed primarily and thus necessitate a return trip to the operating theatre. Infection and loss of function are other concerns associated with this procedure. Here, we present a case of forearm compartment syndrome in the setting of a distal third radius and ulna fracture post trauma. The compartment syndrome was addressed using a tightly-wrapped Esmarch bandage applied from distal to proximal on the patient's upper limb. It was wrapped and unwrapped a number of times until forearm compartment pressures were satisfactory. The patient did not go on to require a fasciotomy.

## Case

**Fig. 1:**
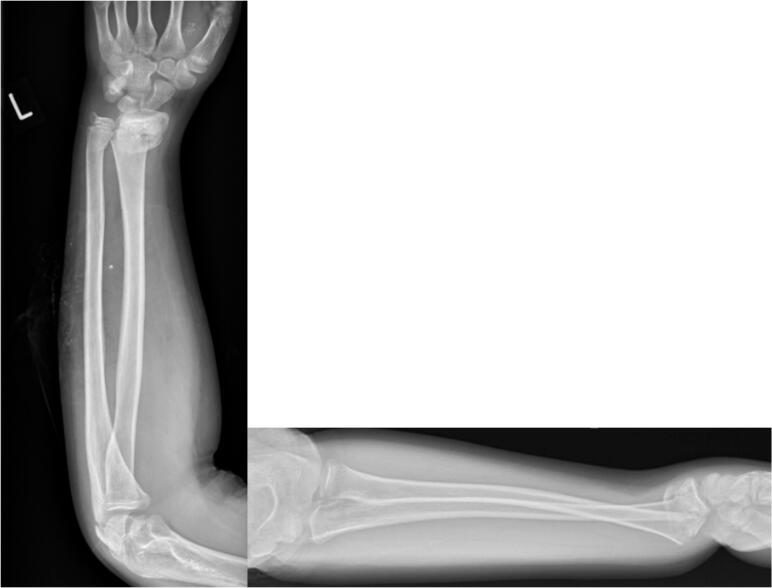
AP and lateral radiograph at initial presentation showing distal third radius & ulna fracture.

A 13 year old boy presented at 11:20 to our emergency department after a fall on to his outstretched left hand from his bicycle. Plain film radiographs were performed and a closed, dorsally angulated left distal radius and ulna fracture (Fig. 1) were noted. A referral was made to our paediatric orthopaedic department and the boy was assessed promptly by the orthopaedic resident. The boy was also found to have concomitant median nerve symptoms, which had begun to develop since the initial referral. The boy had paraesthesia and reduced sensation in his left thumb, index, middle and ring finger. His symptoms were subjectively most troublesome in his middle finger. Both his anterior interosseous nerve (AIN) and posterior interosseous nerve (PIN) were intact. Of note, there was no pain elicited on passive flexion and extension of the boy's fingers and thumb, radial and brachial artery pulses were present and the patient's pain was controlled with routine oral analgesia post fracture. Capillary refill time (CRT) was <2 s. The boy had no other associated injuries. The patient last ate at 08:30 that day and last liquid intake was water taken at 11:30. The patient was told to continue to fast, his upper limb was marked and consent was obtained.

**Fig. 2:**
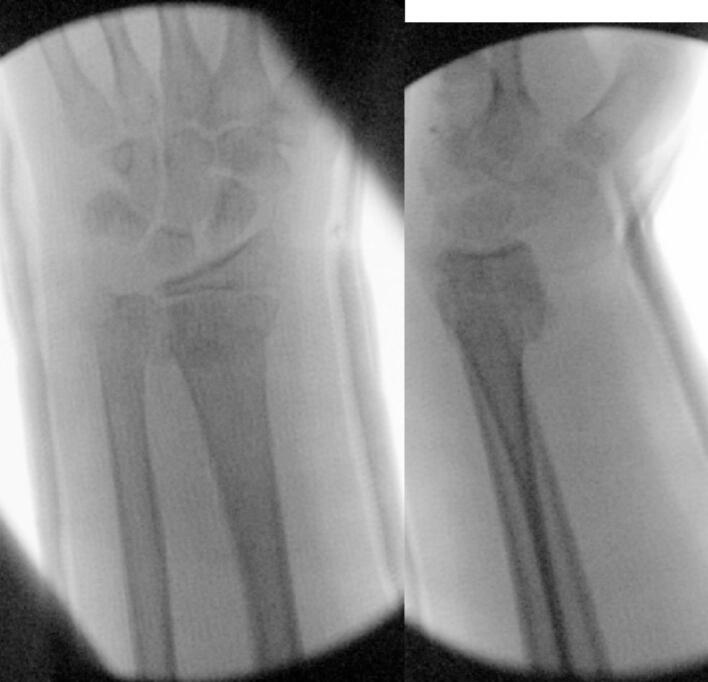
AP and lateral radiograph from intra-operative screening during closed reduction & casting.

At 17:25 the same day, the patient underwent a manipulation under anaesthesia and application of an above elbow cast in the operating theatre under image intensifier guidance. A satisfactory reduction was achieved (Fig. 2) and the patient returned to the ward post operatively at 18:00. The orthopaedic resident was contacted by the ward to review the patient at 21:00 as the boy was complaining of “severe pain and reduced sensation in his fingers”. On arrival, the boy was complaining of 9/10 pain. Significant swelling of the hand and wrist within the cast was noted. His median nerve symptoms had worsened and he was beginning to develop ulnar nerve symptoms. He had decreased sensation in his median nerve distribution, similar to his pre operative findings. The patient was having difficulty with abduction and adduction of his fingers, particularly when compared to his contralateral hand. AIN and PIN were again intact. CRT was again <2 s. His most worrying finding at this time was severe pain on passive flexion and extension of his fingers and thumb. His cast was bi-valved on the ward but provided no relief. Subsequent removal of the cast again provided no relief. At this point, the patient had been given paracetamol, ibuprofen, clonidine and oramorph. His forearm was noted to be tense and a clinical diagnosis of compartment syndrome with suspected acute carpal tunnel syndrome was made. After discussion with the paediatric orthopaedic attending on-call, a decision was made to return the patient to the operating theatre.

A Stryker monitor was used to measure compartment pressures in the left forearm. A compartment pressure of 68 mmHg was noted in the flexor compartment and 16 mmHg in the extensor compartment. An Esmarch bandage was temporarily applied in a retrograde fashion from distal to proximal. The bandage was wrapped tightly around the upper limb and elevated in a similar fashion as when attempting to exsanguinate a limb prior to upper limb surgery. This technique was repeated twice. Compartment pressure was re-checked within the flexor compartment and was now 43 mmHg. The technique was employed a further 3 times and final compartment pressure in the flexor compartment was 23 mmHg. All compartments within the left forearm were now soft on clinical examination.

**Fig. 3:**
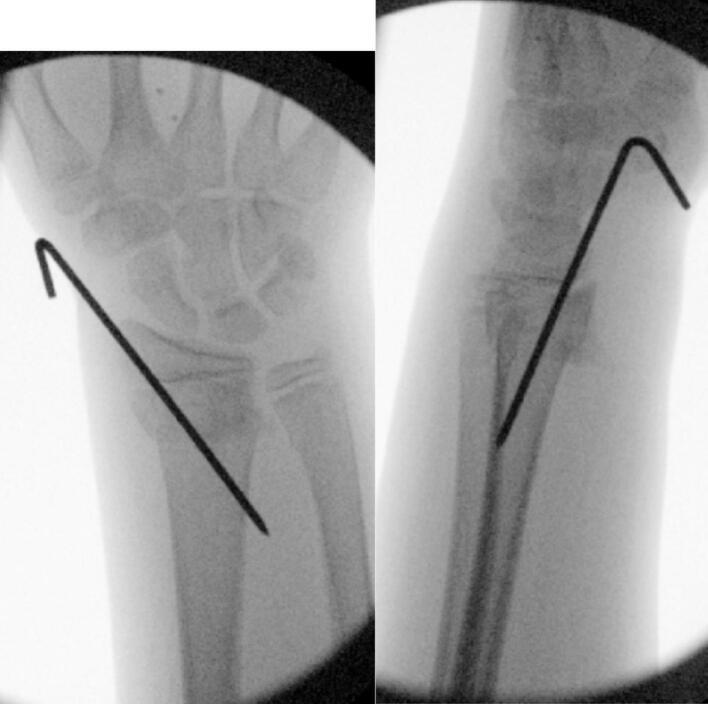
Intra-operative screening from combined carpal tunnel decompression & closed reduction & K-wiring of distal radius fracture.

A decision was made to perform an acute carpal tunnel release and distal radius fixation, but not a fasciotomy, based on the new compartment pressure readings. A tourniquet was applied and the patient was painted and draped in standard sterile fashion. The median nerve was identified and a large haematoma was noted in the carpal tunnel. On release of the transverse carpal ligament, using a McDonald dissector to protect the nerve, a large-sized haematoma was evacuated. This haematoma tracked proximally and distally. A 2 mm stainless steel K-wire was inserted in a retrograde fashion using the radial styloid as the point of entry to fix the distal radius fracture (Fig. 3). A well-padded, below elbow soft cast was applied. The patient returned to the ward at 02:30 and was elevated in a roller towel overnight.

The patient was reviewed again at 06:30 by the orthopaedic resident. His pain was subjectively reported as 2/10, as compared to the 9/10 pre operatively. His median nerve sensation had improved. Thumb sensation was normal, with reduced sensation only in the distal phalanx of index and middle fingers. Ulnar nerve sensation was intact. He still had some difficulty with finger abduction and adduction. AIN and PIN were intact. His hand was warm, pink and perfused with a CRT of <2 s. The boy was kept in hospital for a further 24 h of observation, by which time, all symptoms had resolved.

**Fig. 4:**
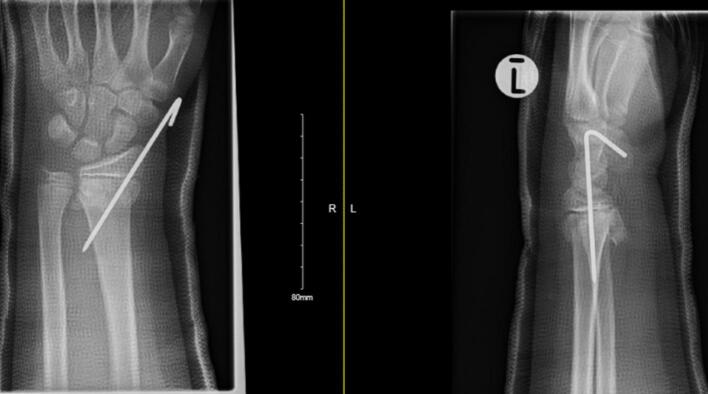
AP and lateral radiographs 5 days post-operatively.

**Fig. 5:**
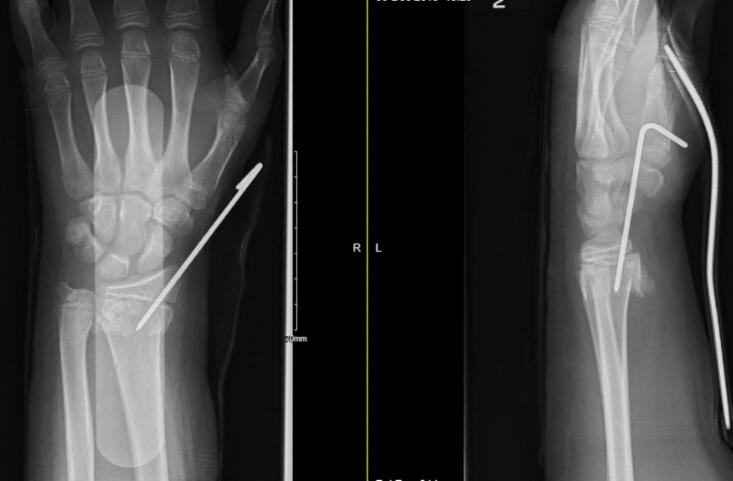
AP and lateral radiograph showing backing out of K-wire.

**Fig. 6:**
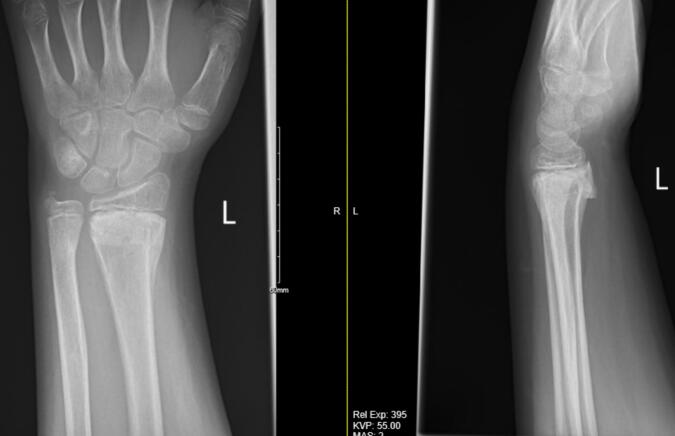
Final radiographs at 8 weeks showing healed radius & ulna fractures.

He had subsequent follow-up in the fracture clinic 5 days later and his radiograph showed acceptable fracture alignment (Fig. 4). A later fracture clinic appointment revealed that the boy's K-wire had backed out (Fig. 5) but the fracture was in an acceptable position, thus the K-wire was removed. Final radiographs taken 8 weeks post operatively showed healed fractures of the radius & ulna (Fig. 6). He had full active and passive range of motion of his left wrist on examination. The boy remained asymptomatic throughout follow-up from a neurological perspective and was subsequently discharged from the fracture clinic.

## Discussion

Compartment syndrome is a surgical emergency and poses a risk to the viability of a limb without urgent treatment (1). We have presented a non-operative technique for the treatment of upper limb compartment syndrome in the setting of an underlying fracture; one such case does not exist in the published literature. We did however note where the technique was described in the successful treatment of an upper limb compartment syndrome, but not involving a fracture. This case involved a patient undergoing elective cardiac surgery who required a rapid infusion of IV fluids due to low intravascular volume. Unfortunately, the patient developed an acute compartment syndrome of the right forearm due to acute venous obstruction caused by extravasation of fluid (2). In the same manner as how our patient was treated, the arm was elevated 90 degrees and an Esmarch bandage applied, wrapped tightly from fingers to axilla. The process was repeated 5 times and there was a decrease in the tension and cyanosis of the affected forearm. With 10 more applications, both radial and ulnar pulses became palpable with the skin turning pink and an improvement in temperature. Compartment pressures improved and the patient was also spared from a fasciotomy (2). Interestingly, the authors did not recommend the use of the technique in the setting of fracture. This perhaps adds more weight to the significance of our success in managing this fractured patient without a formal fasciotomy.

This poses the question as to whether fasciotomies can be avoided in compartment syndrome of a forearm, at least in a paediatric cohort with a distal radius and ulna fracture? It is well established that formal volar and extensor +/− mobile wad fasciotomies are the gold standard for upper limb compartment syndrome (3). The Esmarch bandage is a versatile piece of equipment, even proving useful in maintenance of reduction in certain fracture patterns (4). By applying an Esmarch bandage in a retrograde fashion, it is theoretically possible to cause backflow of the oedema and blood flow to reduce intra compartmental pressure, as seen in our patient. Crucially, we were able to demonstrate a clear and objective improvement in the patient's clinical status. This was achieved through serial clinical examinations of the forearm as well as repeated compartment pressure measurements. Our technique may only be adopted where strong clinical judgment is also exercised as the potential for morbidity and even mortality from a missed or delayed compartment syndrome is significant (1).

We are not advocating that all compartment syndromes of the forearm be treated in this manner, however. Fasciotomy should remain the gold standard treatment and all involved in treating the patient with suspected or confirmed compartment syndrome should be prepared to perform this procedure. One of the cardinal signs of compartment syndrome is pain, often described as “pain out of proportion to the injury”. This can be difficult to assess in the paediatric population (5). The three As (anxiety, agitation and analgesic requirement) have been more recently described in the paediatric literature as allowing earlier detection (6). Certainly, our patient was anxious and distressed on the ward, prompting multiple clinical reviews. His analgesic requirements increased steadily too until they were completely ineffective. Prompt diagnosis and treatment with fasciotomy in children results in excellent long-term outcomes (5). As compartment syndrome is generally very painful, we would only recommend the use of our Esmarch bandage technique in the anaesthetised patient in an operating theatre and in whom a diagnosis of compartment syndrome is suspected/confirmed and a potential fasciotomy is imminent. In terms of a strict cut-off time after which the Esmarch bandage technique may not be successful, we cannot say for sure. Our technique has not been employed in a sufficiently varied cohort of patients, in terms of time from presentation to time of application, to strongly suggest a cut off time. The technique was devised by the senior author and is not necessarily a standard, widespread technique within our department. This case report serves to highlight the improvement that this particular patient experienced after this last-minute intervention, in the context of a clinical decision having been made to bring the patient to theatre for fasciotomy. We would like to strongly emphasise that the Esmarch bandage technique described here be used as an adjunct to traditional management of compartment syndrome. Hence, it should only be employed in those patients where a decision has been made to operate and should not delay this critical step in care. In this scenario, if there is any doubt about an improvement in the patient's clinical exam and compartment measurements with the technique, it should be abandoned and prompt fasciotomy performed.

We acknowledge that the carpal tunnel decompression played a role in the resolution of some of the patient's symptoms. We had, however, already objectively proved a resolution in the patient's compartment syndrome with formal, repeated compartment pressure measurements. The ability to avoid a formal fasciotomy in a 13 year old child cannot be understated. Fasciotomies, although effective, can be associated with significant morbidity including scars, infection, skin grafting, and psychological effects (7). Our conservative technique is easily-applied and may work in reducing compartment pressure and restoring circulation. It can be applied quickly and, crucially, does not cause any further harm to the patient.

## Conclusion

We have described a successful case of treating forearm compartment syndrome in the setting of a paediatric forearm fracture conservatively, without the need for a formal fasciotomy. We demonstrated an objective improvement in compartment pressures with repeated applications of the Esmarch bandage technique. It is quick to implement and safe for the patient. We advocate its use in those patients where a fasciotomy is already planned as that remains the gold standard treatment. This technique should be used to potentially avoid a fasciotomy and the subsequent morbidity associated with that surgical procedure. It should be used in conjunction with sound clinical judgment and examination technique.

## CRediT authorship contribution statement

**Ben Murphy:** Writing – review & editing, Writing – original draft, Formal analysis, Data curation, Conceptualization. **Patrick Carroll:** Writing – review & editing, Writing – original draft, Formal analysis, Data curation, Conceptualization. **Jacques Noel:** Supervision, Formal analysis, Data curation, Conceptualization.

## Funding

This research did not receive any specific grant from funding agencies in the public, commercial, or not-for-profit sectors.

## Declaration of competing interest

The authors declare that they have no known competing financial interests or personal relationships that could have appeared to influence the work reported in this paper.
